# Comprehensive analysis of immune cell enrichment in the tumor microenvironment of head and neck squamous cell carcinoma

**DOI:** 10.1038/s41598-021-95718-9

**Published:** 2021-08-09

**Authors:** Ikko Mito, Hideyuki Takahashi, Reika Kawabata-Iwakawa, Shota Ida, Hiroe Tada, Kazuaki Chikamatsu

**Affiliations:** 1 Department of Otolaryngology-Head and Neck Surgery, Gunma University Graduate School of Medicine, 3-39-22, Showa-machi, Maebashi, Gunma 371-8511 Japan; 2 Division of Integrated Oncology Research, Gunma University Initiative for Advanced Research, 3-39-22, Showa-machi, Maebashi, Gunma 371-8511 Japan

**Keywords:** Immunology, Tumour immunology

## Abstract

Head and neck squamous carcinoma (HNSCC) is highly infiltrated by immune cells, including tumor-infiltrating lymphocytes and myeloid lineage cells. In the tumor microenvironment, tumor cells orchestrate a highly immunosuppressive microenvironment by secreting immunosuppressive mediators, expressing immune checkpoint ligands, and downregulating human leukocyte antigen expression. In the present study, we aimed to comprehensively profile the immune microenvironment of HNSCC using gene expression data obtained from public database. We calculated enrichment scores of 33 immune cell types based on gene expression data of HNSCC tissues and adjacent non-cancer tissues. Based on these scores, we performed non-supervised clustering and identified three immune signatures—cold, lymphocyte, and myeloid/dendritic cell (DC)—based on the clustering results. We then compared the clinical and biological features of the three signatures. Among HNSCC and non-cancer tissues, human papillomavirus (HPV)-positive HNSCCs exhibited the highest scores in various immune cell types, including CD4+ T cells, CD8+ T cells, B cells, plasma cells, basophils, and their subpopulations. Among the three immune signatures, the proportions of HPV-positive tumors, oropharyngeal cancers, early T tumors, and N factor positive cases were significantly higher in the lymphocyte signature than in other signatures. Among the three signatures, the lymphocyte signature showed the longest overall survival (OS), especially in HPV-positive patients, whereas the myeloid/DC signature demonstrated the shortest OS in these patients. Gene set enrichment analysis revealed the upregulation of several pathways related to inflammatory and proinflammatory responses in the lymphocyte signature. The expression of *PRF1*, *IFNG*, *GZMB*, *CXCL9*, *CXCL10*, *PDCD1*, *LAG3*, *CTLA4*, *HAVCR2*, and *TIGIT* was the highest in the lymphocyte signature. Meanwhile, the expression of PD-1 ligand genes *CD274* and *PDCD1LG2* was highest in the myeloid/DC signature. Herein, our findings revealed the transcriptomic landscape of the immune microenvironment that closely reflects the clinical and biological significance of HNSCC, indicating that molecular profiling of the immune microenvironment can be employed to develop novel biomarkers and precision immunotherapies for HNSCC.

## Introduction

Head and neck squamous cell carcinoma (HNSCC) is the sixth most common malignant tumor worldwide^1,2^. In addition to tobacco-derived carcinogens and excessive alcohol consumption, infection with oncogenic strains of human papillomavirus (HPV) has been recognized as a major risk factor for developing HNSCCs, mainly oropharyngeal cancers^3,4^. Despite ongoing improvements in therapeutic strategies, including surgery, chemotherapy, and radiotherapy, the 5-year survival rate remains 66%. Recently, cancer immunotherapy has been developed as an additional approach for various cancer types, including HNSCC^5–7^. The programmed cell death 1/programmed cell death ligand 1 (PD-1/PD-L1) axis is a crucial target for immune checkpoint therapies^8^. In clinical settings, anti-PD-1 antibodies have been widely employed for treating recurrent or metastatic HNSCC; however, survival benefits have been observed in only 20–30% of patients. Accordingly, novel biomarkers have been widely investigated to improve the efficacy of immunotherapies.

In the tumor microenvironment (TME), various stromal cells such as immune cells, fibroblasts, and endothelial cells exist and interact with tumor cells^9,10^. HSNCC is highly infiltrated by immune cells, including tumor-infiltrating lymphocytes (TILs) and myeloid lineage cells^11,12^. In the TME of HNSCC, tumor cells reportedly orchestrate a highly immunosuppressive state by secreting immunosuppressive mediators, expressing immune checkpoint ligands, and downregulating human leukocyte antigen expression^13,14^. These tumor cell behaviors result in the dysfunction and exhaustion of cytotoxic T lymphocytes (CTLs), as well as increased infiltration and activation of immunosuppressive cell types, such as regulatory T cells (Tregs), tumor-associated macrophages, and myeloid-derived suppressor cells (MDSCs)^15^. As immune checkpoint agents target the interaction between tumor cells and immune cells, a comprehensive analysis of the complex state of the immune microenvironment would be beneficial for developing new biomarkers and precision immunotherapies.

In the present study, we aimed to comprehensively profile the immune microenvironment of HNSCC using gene expression data obtained from public database. We calculated the cell enrichment scores of 33 immune cell types based on RNA-seq data of both HNSCC tissues and adjacent non-cancer tissues. Based on these scores, we performed non-supervised clustering and identified three immune signatures—cold, lymphocyte, and myeloid/dendritic cell (DC)—based on clustering results. Finally, the clinical and biological features of the three signatures were compared.

## Results

### HPV-positive HNSCCs exhibited upregulated enrichment of various immune cells

**Figure 1:**
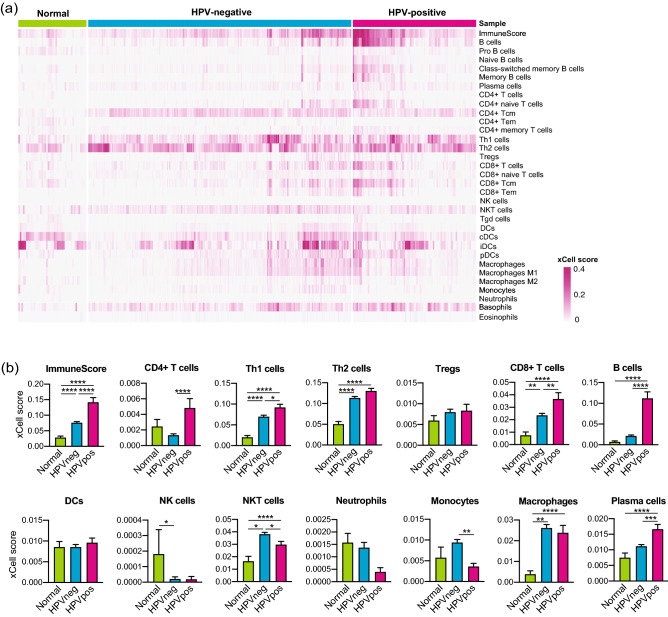
Enrichment scores of 33 immune cell types in normal tissues and HNSCCs. (**a**) Heat map of xCell enrichment scores of 33 immune cell types in 44 normal tissues, 97 HPV-positive HNSCCs, and 423 HPV-negative HNSCCs of TCGA cohort. (**b**) Bar graphs of enrichment scores of major immune cell types shown in (**a**). *HNSCC* Head neck squamous cell carcinoma, *HPV* Human papillomavirus, *HPVneg* HPV-negative, *HPVpos* HPV-positive, *DC* Dendritic cells. *, *P* < 0.05; **, *P* < 0.01; ***, *P* < 0.001; ****, *P* < 0.0001.

We calculated the enrichment scores of 33 immune cell types among 520 HNSCCs and 44 normal samples (Fig. 1, Suppl. Fig. 1) in the TCGA cohort. HPV-positive HNSCCs exhibited the highest scores for various immune cell types, including CD4+ T cells, CD8+ T cells, B cells, plasma cells, basophils, as well as their subpopulations. Normal samples exhibited the lowest scores for several cell types. Similarly, we calculated the enrichment scores among 270 HNSCCs in the GSE65858 cohort (Suppl. Fig. 3). In consistent with the TCGA cohort, HPV-positive tumors exhibited higher scores for various immune cell types than HPV-negative tumors.

### The lymphocyte signature correlated with clinical parameters and better prognosis

**Figure 2:**
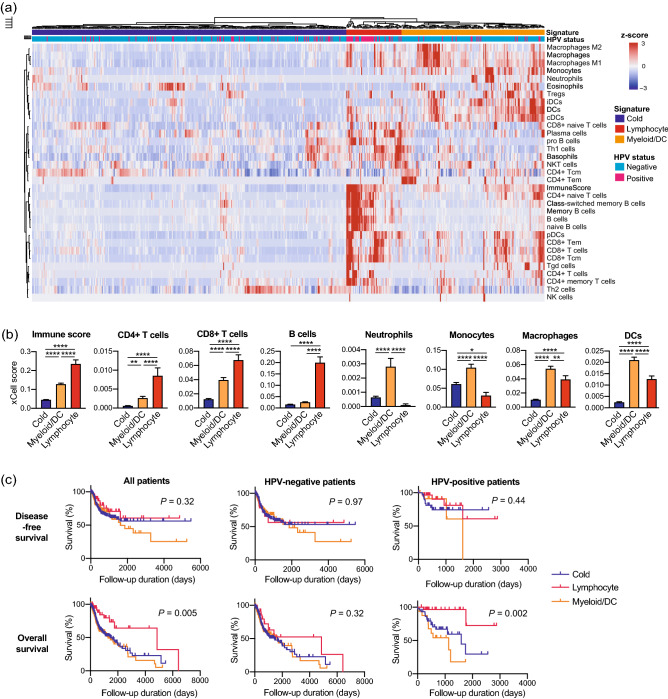
The lymphocyte signature correlates with a favorable prognosis. (**a**) Heat map showing non-supervised hierarchical clustering of 520 HNSCCs of TCGA cohort based on enrichment scores of 33 immune cell types. (**b**) Bar graphs of enrichment scores upregulated in the lymphocyte signature or myeloid/DC signature. (**c**) Kaplan–Meier survival curves based on the three immune signatures. Disease-free survival was evaluated in all patients (n = 429), HPV-negative patients (n = 348), and HPV-positive patients (n = 81). Overall survival was evaluated in all patients (n = 495), HPV-negative patients (n = 403), and HPV-positive patients (n = 92). *HNSCC* Head neck squamous cell carcinoma, *HPV* Human papillomavirus, *DC* Dendritic cells. *, *P* < 0.05; **, *P* < 0.01; ****, *P* < 0.0001.

Based on hierarchical clustering results, we segregated 520 HNSCCs in the TCGA cohort into three immune signatures (Fig. 2a). The lymphocyte signature was characterized by the enrichment of CD4+ T cells, CD8+ T cells, B cells, and plasma cells (Fig. 2b, Suppl. Fig. 2). The myeloid/DC signature exhibited enrichment of neutrophils, macrophages, monocytes, DCs, Tregs, and eosinophils (Fig. 2b, Suppl. Fig. 2). Table 1 represents correlations between the immune signatures and clinical parameters. The proportion of HPV-positive patients (59%) was significantly higher in the lymphocyte signature than in other signatures. Regarding primary lesions, the proportion of the oropharynx (50%) was higher in the lymphocyte signature than in other signatures. The proportion of patients with early T factor (63%) was higher in the lymphocyte signature than in other signatures. Furthermore, the proportion of N factor-positive patients (61%) was higher in the lymphocyte signature than in other signatures. No difference was observed between the immune signatures and the M factor/tumor-node-metastasis (TNM) stage. Univariate survival analyses revealed that the lymphocyte signature showed the longest overall survival (OS) among the three signatures, especially in HPV-positive patients (Fig. 2c). The myeloid/DC signature showed the shortest OS among the three signatures in HPV-positive patients. No difference in disease-free survival (DFS) was observed between the immune signatures. Multivariate regression analyses revealed that the lymphocyte signature was an independent prognostic factor for better OS (Table 2).

**Table 1:** Relationship between immune cell signature and clinical parameters in 520 patients with HNSCC.

Variables	Immune cell signature			
	Cold (n = 319)	Myeloid/DC (n = 145)	Lymphocyte (n = 56)	*p* value
**HPV status**				
Negative	271	129	23	< 0.0001
Positive	48	16	33	
**Primary lesion**				
Hypopharynx	6	3	1	< 0.0001
Larynx	86	24	6	
Oral cavity	210	113	28	
Oropharynx	17	5	21	
**T factor**				
T0-2	120	55	35	0.002
T3-4	199	90	21	
**N factor**				
Negative	143	57	13	0.0005
Positive	165	79	34	
Unknown	11	9	9	
**M factor**				
M0	307	142	55	0.582
M1	3	1	1	
Unknown	9	2	0	
**TNM stage**				
I-II	67	33	11	0.86
III-IV	252	112	45	

*HNSCC* Head and neck squamous cell carcinoma, *HPV* Human papillomavirus, *TNM* Tumor-node-metastasis, *DC* Dendritic cell.

**Table 2:** Univariate and multivariate survival analyses of OS and DFS in HNSCC patients.

Variables	Disease free survival			Overall survival		
	Univariate	Multivariate		Univariate	Multivariate	
	*p* value	HR (95% CI)	*p* value	*p* value	HR (95% CI)	*p* value
**HPV status**						
Negative	0.027	1	0.037	0.132		
Positive		0.566 (0.331–0.967)				
**Primary lesion**						
Hypopharynx	0.158			0.140		
Larynx						
Oral cavity						
Oropharynx						
**T factor**						
T0-2	0.001	1	0.132	0.0002	1	0.016
T3-4		1.474 (0.889–2.444)			1.839 (1.118–3.026)	
**N factor**						
Negative	0.06			0.037	1	0.217
Positive					1.267 (0.870–1.846)	
**M factor**						
M0	0.26			0.001	1	0.0005
M1					6.263 (2.215–17.707)	
**TNM stage**						
I–II	0.009	1	0.254	0.009	1	0.483
III–IV		1.457 (0.763–2.782)			0.788 (0.405–1.534)	
**Immune signature**						
Cold	0.320			0.005	1	
Lymphocyte					0.362 (0.172–0.763)	0.008
Myeloid/DC					1.077 (0.780–1.486)	0.654

*DFS* Disease free survival, *OS* Overall survival, *HNSCC* Head and neck squamous cell carcinoma, *HR* Hazard ratio, *CI* Confidence interval, *DC* Dendritic cell.

Alternatively, we performed hierarchical clustering of the GSE65858 cohort and confirmed that HNSCCs cases were also divided into three immune signatures (Suppl. Fig. 4a,b). In addition, correlations between the immune signatures and clinical parameters, including HPV status and primary lesions, exhibited same trends as the TCGA cohort (Suppl. Table 1). Although survival analyses showed no significant difference between the immune signatures, the myeloid/DC signature tended to correlate with shorter OS (Suppl. Fig. 4c). Moreover, in HPV-negative patients, the myeloid/DC signature significantly correlated with shorter DFS and OS.

### The lymphocyte signature correlated with upregulated inflammatory pathways

**Figure 3:**
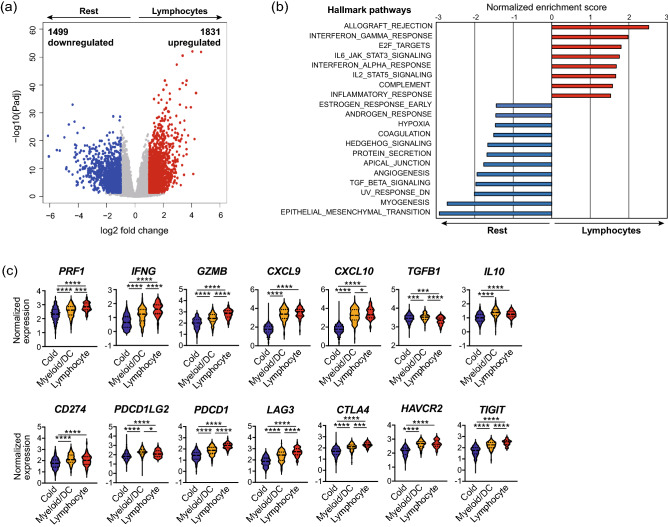
The lymphocyte signature correlated with activated cytotoxic T cell response. (**a**) Volcano plot of differentially expressed genes in the lymphocyte signature of TCGA cohort. Red dots represent upregulated genes (Padj < 0.05, log2FC > 1), whereas blue dots represent downregulated genes (Padj < 0.05, log2FC <  − 1). (**b**) Upregulated and downregulated hallmark pathways in the lymphocyte signature obtained by GSEA (FDR < 0.05). (**c**) Violin plots of normalized expression of immune-related genes. *GSEA* Gene set enrichment analysis, *FDR* False discovery rate. *, *P* < 0.05; ***, *P* < 0.001; ****, *P* < 0.0001.

Based on these findings, we then focused on the transcriptomic significance of the lymphocyte signature. In the TCGA cohort, 3330 differentially expressed genes (DEGs), including 1831 upregulated and 1499 downregulated genes, were identified in the lymphocyte signature (Fig. 3a, Suppl. Table 2). Additionally, we performed gene set enrichment analysis (GSEA) to identify pathways upregulated in the lymphocyte signature (Fig. 3b). In the lymphocyte signature, 8 hallmark pathways were upregulated, whereas 12 were downregulated (false discovery rate ˂ 0.05). Several pathways associated with inflammatory and proinflammatory responses, such as allograft rejection, interferon (IFN) gamma response, interleukin (IL) 6-Janus kinase (JAK)-signal transducer and activator of transcription (STAT) 3 signaling, interferon-alpha response, IL2 STAT5 signaling, and complement, were upregulated in the lymphocyte signature. Meanwhile, several pathways representing malignant features of HNSCCs, including hypoxia, angiogenesis, transforming growth factor (TGF)-β signaling, and epithelial-mesenchymal transition, were downregulated in the lymphocyte signature. Similarly, we also performed GSEA with the GSE65858 cohort, confirming the upregulation of similar pathways in the lymphocyte signature (Suppl. Fig. 5a).

### The lymphocyte signature correlated with activated cytotoxic T cell response

We investigated the expression of immune-related genes to compare cytotoxic T cell responses across the three immune signatures (Fig. 3c). The lymphocyte signature demonstrated the highest expression of genes related to cytotoxic T cell responses, including *PRF1*, *IFNG*, *GZMB, CXCL9,* and *CXCL10*. Additionally, the expression of immune checkpoint genes, including *PDCD1*, *LAG3*, *CTLA4*, *HAVCR2*, and *TIGIT*, was the highest in the lymphocyte signature. Meanwhile, the expression of PD-1 ligand genes *CD274* and *PDCD1LG2* was the highest in the myeloid/DC signature, which also revealed the highest expression of immunosuppressive genes, *TGFB1* and *IL10*. Similarly, we confirmed these results in the GSE65858 cohort (Suppl. Fig. 5b).

## Discussion

Recent advances in bioinformatics and the accumulation of public genomic databases have enabled the comprehensive genomic characterization of cancers in a large cohort^16^. In the present study, we elucidated the transcriptomic landscape of the immune microenvironment that closely reflects the clinical and biological significance of HNSCC. Our results suggest that molecular profiling of the immune microenvironment can potentially help develop new biomarkers and precision immunotherapies.

The comparison of immune cell enrichment scores revealed high infiltration of various immune cells into HNSCC tissues, especially in HPV-positive HNSCCs. The scores of various TILs were higher in HNSCCs than in normal tissues. Among TILs, CD8+ T cells are the main subset of CTLs and play vital roles in tumor eradication. CD8+ T cells, as well as their subset CD8+ central memory T cells (Tcm) and CD8+ effector memory T cells (Tem), represented significantly higher scores in HPV-positive HNSCCs than in other groups, indicating a highly activated CTL function in the TME of HPV-positive HNSCCs^17,18^. Consistent with our results, accumulating evidence suggests that HPV-positive HNSCCs correlate with T cell-enriched TME, increased T cell receptor pathway signaling, activated cytotoxic capacity, and viral antigen-specific CD8+ T cell infiltration into the TME^19–21^. Additionally, we observed a significant increase in B cell subsets and plasma cells in HPV-positive tumors but not in HPV-negative tumors. Although the significance of B cell infiltration in the TME is not well understood, recent studies have reported the anti-tumor activity of B cells and plasma cells through antigen presentation and antibody production^22^. A recent study reported the presence of HPV-specific antibody-secreting cells in the TME of HPV-positive tumors^23^. Moreover, Kim et al. have reported that B cells correlate with longer OS and are activated by radiation and PD-1 blockade therapy^24^. These findings suggest the potential of B-cell-targeted immunotherapy. Further investigations regarding the specific roles of B cells and plasma cells in the TME are warranted.

Herein, non-supervised clustering of HNSCC cases based on the cell enrichment scores of 33 immune cell types revealed three immune signatures: cold, lymphocyte, and myeloid/DC. The lymphocyte signature correlated with the HPV-positive type, early T factor, positive N factor, and favorable prognosis in the TCGA cohort. Notably, the presence of T cell subsets has been widely investigated in several malignancies^25–29^. In HNSCC, the presence of TILs in the TME is reportedly considered a favorable prognostic factor^30,31^. Moreover, Tsujikawa et al. have previously assessed immune cell complexity profiles of 38 HNSCC cases using multiplex immunohistochemistry^32^. They acquired cell densities of 15 immune cell lineages using image cytometry, followed by normalization and unsupervised hierarchical clustering. Their analysis revealed three immune signatures: lymphoid-inflamed, myeloid-inflamed, and hypo-inflamed. The myeloid-inflamed signature exhibited significantly shorter OS. In addition, the lymphoid-inflamed signature consisted of more HPV-positive HNSCCs than the other signatures. Surprisingly, the results of the present study are consistent with those of their protein expression-based analysis. Although bulk RNA sequencing cannot evaluate the localization of each immune cell in the TME, our results suggest that bulk RNA sequencing-based molecular profiling has the potential to comprehensively profile immune cell complexity of the TME in combination with protein expression-based profiling, such as multiplex IHC. In addition to Tsujikawa’s work, our results of the TCGA cohort revealed that the myeloid/DC signature dramatically correlates with shorter OS in HPV-positive HNSCCs but not in HPV-negative cases. HPV-positive HNSCCs are widely recognized to exhibit a better prognosis than HPV-negative HNSCCs^33,34^. However, in clinical settings, some HPV-positive HNSCCs present aggressive behavior, resulting in a poor prognosis. Therefore, biomarkers that indicate the aggressive phenotype of HPV-positive HNSCCs are needed. The screening for myeloid-enriched TME has the potential to predict survival and allow precision medicine in HPV-positive HNSCCs.

We further focused on the transcriptomic significance of immune signatures. GSEA revealed the upregulation of multiple pathways related to inflammatory and proinflammatory responses, as well as the downregulation of pathways closely related to cancer hallmarks in the lymphocyte signature, as shown in Fig. 3b and Suppl. Fig. 5a. The upregulation of IFN-α responses, IFN-γ, and IL2 STAT5 signaling represents activated CTL responses, consistent with the presence of abundant lymphocytes. Furthermore, the lymphocyte signature showed the highest expression of both cytotoxic response-related genes *PRF1*, *IFNG*, *GZMB*, *CXCL9*, and *CXCL10*, and immune checkpoint genes *PDCD1*, *LAG3*, *CTLA4*, *HAVCR2*, and *TIGIT*. As these immune checkpoint molecules reportedly function as receptors for T cell inactivation and exhaustion signals, these molecules are abundantly expressed on effector memory T cells and tissue-resident memory T cells, which are activated phenotypes of T cells^35–38^. Accordingly, PD-1-expressing TILs are reportedly considered a favorable prognostic biomarker in HPV-positive HNSCCs^39^. Overall, the lymphocyte signature represented the enrichment of lymphocyte infiltration, activation of CTL functions, and favorable prognosis. As the cost of RNA sequencing has recently decreased, molecular profiling of the immune microenvironment using biopsy tissues may provide an alternative for the initial diagnosis of HNSCCs. However, in bulk RNA sequencing, the localization of immune cells cannot be determined. Dual profiling using both molecular and protein-based profiling would be helpful in comprehensively profiling the complexity of the immune milieu of the TME.

In conclusion, the present study revealed the transcriptomic landscape of the immune microenvironment that closely reflects the clinical and biological significance of HNSCC. Our results suggest that molecular profiling of the immune microenvironment can be employed for developing new biomarkers and precision immunotherapies for HNSCC.

## Materials and methods

### Acquisition of the cancer genome atlas (TCGA) data

RNA-seq data (Illumina Hiseq RNAseq V2, raw counts, and normalized counts) and clinical data were obtained from TCGA Research Network (TCGA Provisional version updated in 2016, http://cancergenome.nih.gov/). In total, 564 cases, consisting of 44 normal samples, 97 HPV-positive HNSCCs, and 423 HPV-negative HNSCCs, were included. Alternatively, GSE65858 dataset, including microarray data (Illumina HumanHT-12 V4.0 expression beadchip platform) and clinical data, were obtained from the Gene Expression Omnibus (GEO) database. In total, 270 cases, consisting of 73 HPV-positive HNSCCs, 196 HPV-negative HNSCCs, and 1 HPV-unknown HNSCC were included.

### Cell type enrichment analysis

We performed cell type enrichment analysis to evaluate the enrichment of 33 immune cell types in both TCGA dataset and GSE65858 dataset using the xCell tool^40^. Enrichment scores were calculated using the xCell R package, version 1.1.0. The calculated scores were visualized using the pheatmap R package, version 1.10.12. Then, normal and HNSCC tissue scores were compared.

### Non-supervised hierarchical clustering of HNSCC samples

HNSCC cases underwent non-supervised hierarchical clustering based on cell enrichment scores of 33 immune cell types. Patients were then divided into three immune signatures—cold, lymphocyte, and myeloid/DC—using the cutree R function based on the clustering results. The three signatures were compared in terms of clinical parameters, including HPV status, primary lesion, T factor, N factor, M factor, TNM stage, DFS, and OS. The three immune signatures were also compared to the normalized gene expression of various immune-related genes.

### Differentially expressed gene analysis

In the TCGA cohort, we identified DEGs between the lymphocyte signature group and other signature groups, using the ExperimentHub R package version 1.16.0 and DESeq2 R package version 1.30.0. DEGs were filtered using the threshold |log_2_FC|≥ 1 and an adjusted *p* value of < 0.05. Volcano plots were constructed to visualize DEGs using the calibration R package version 1.7.7.

### Gene set enrichment analysis

GSEA (GSEA v4, Broad Institute) was performed to identify pathways upregulated in the lymphocyte group when compared with other groups. For each gene set, the normalized enrichment score, *p* value, and false discovery rate (FDR) q-values were calculated based on the Hallmark pathway database.

### Statistical analysis

Data were analyzed using R (version 4.0.3; The R Foundation for Statistical Computing, Vienna, Austria) in combination with R studio version 1.3.1093 (R studio, Boston, MA, USA) and GraphPad Prism version 8 (GraphPad Software, San Diego, CA, USA). Student’s t-test and one-way ANOVA with Tukey’s post-hoc test for multiple pairwise testing were employed to compare continuous variables between groups. The Chi-square test for independence and Fisher’s exact test were used for comparing categorical variables. Two-sided *p* values of < 0.05 were considered statistically significant. Survival curves were calculated using the Kaplan–Meier method and compared using the log-rank test. Multivariate regression analysis was performed using the Cox proportional hazards model. Variables were included in subsequent multivariate analyses when *p* values were < 0.05 in univariate analyses.

## Supplementary Information

Supplementary Figures.

Supplementary Tables.
