# Supplementary material for: Comprehensive analysis of immune cell enrichment in the tumor microenvironment of head and neck squamous cell carcinoma
Source: Sci Rep. 2021 Aug 9;11:16134. doi: 10.1038/s41598-021-95718-9 (PMC8352955; doi:10.1038/s41598-021-95718-9)
Supplement: Supplementary file 1 — Supplementary Figures. [file 41598_2021_95718_MOESM1_ESM.pdf]

Suppl.Figure 1

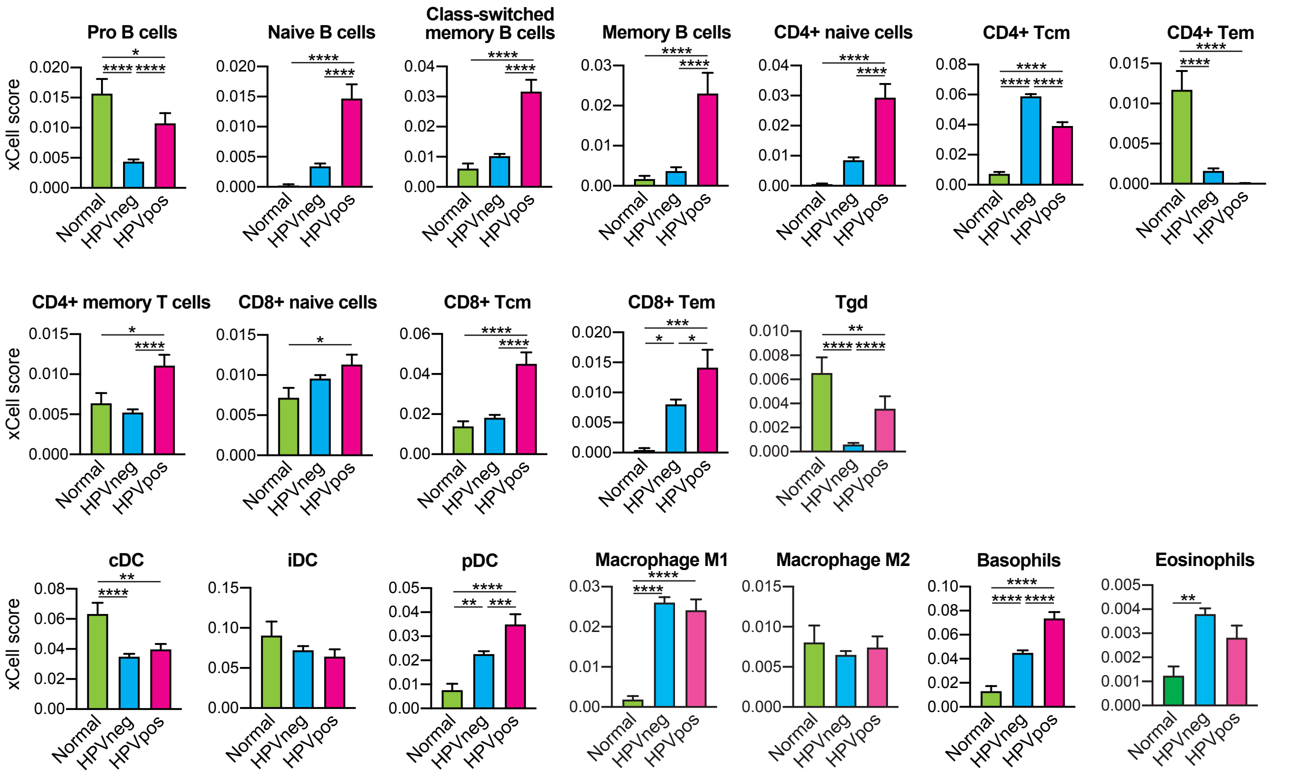

Suppl. Figure 1. Enrichment scores of various immune cell types in normal tissues and HNSCCs

Bar graphs of enrichment scores of immune cell types that are shown in Figure 1a. HPV, human papillomavirus; HPVneg, HPV-negative; HPVpos, HPV-positive; DC, dendritic cells *, $p < 0.05$; **, $p < 0.01$; ***, $p < 0.001$; ****, $p < 0.0001$.

Suppl.Figure 2

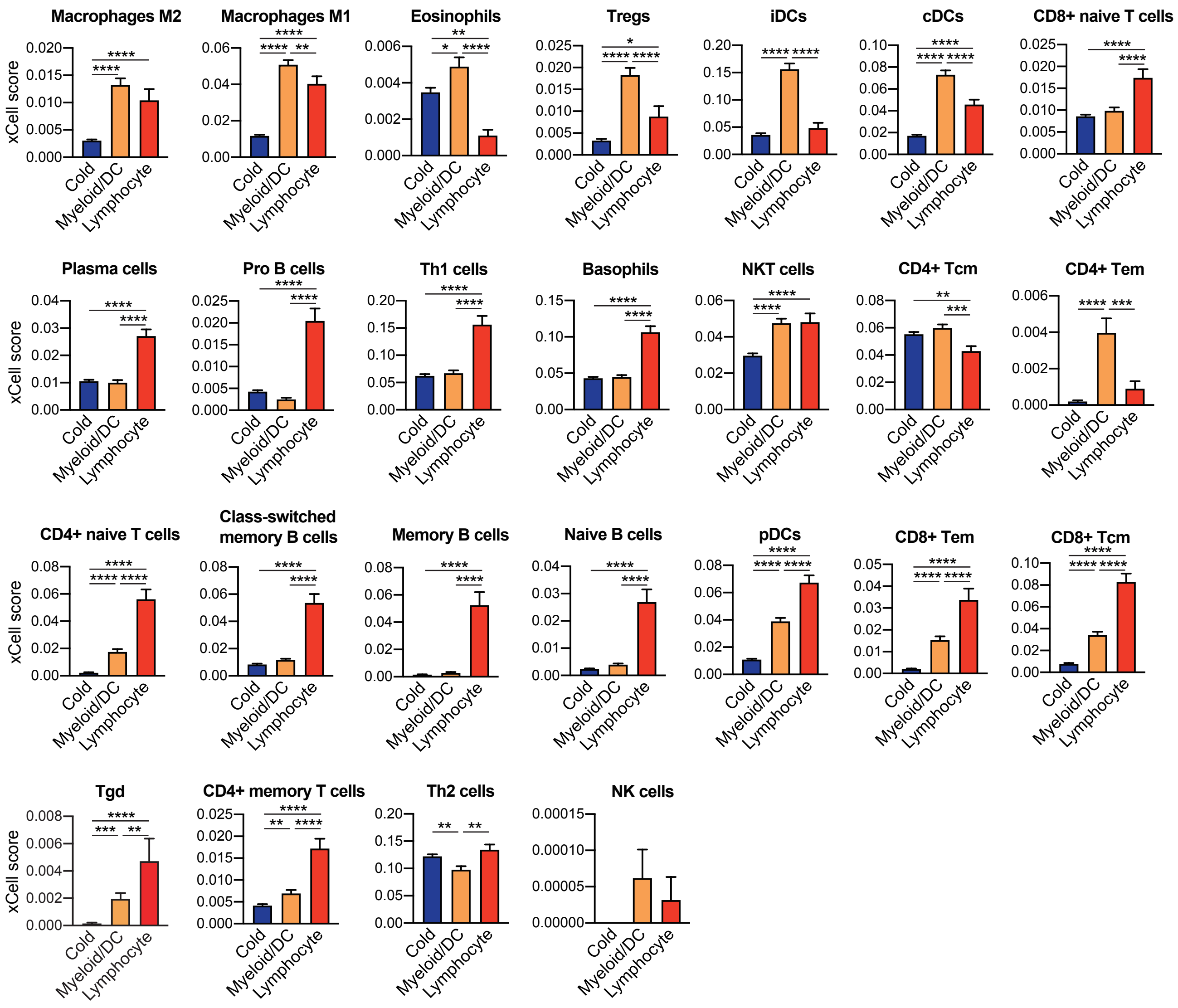

Suppl. Figure 2. Enrichment scores of immune cell types upregulated in the lymphocyte signature or myeloid/DC signature

Bar graphs of enrichment scores of immune cell types upregulated in the lymphocyte signature or myeloid/DC signature (additional data to Figure 2b). P < 0.05; **, P < 0.01; ***, P < 0.001; ****, P < 0.0001.

Suppl.Figure 3

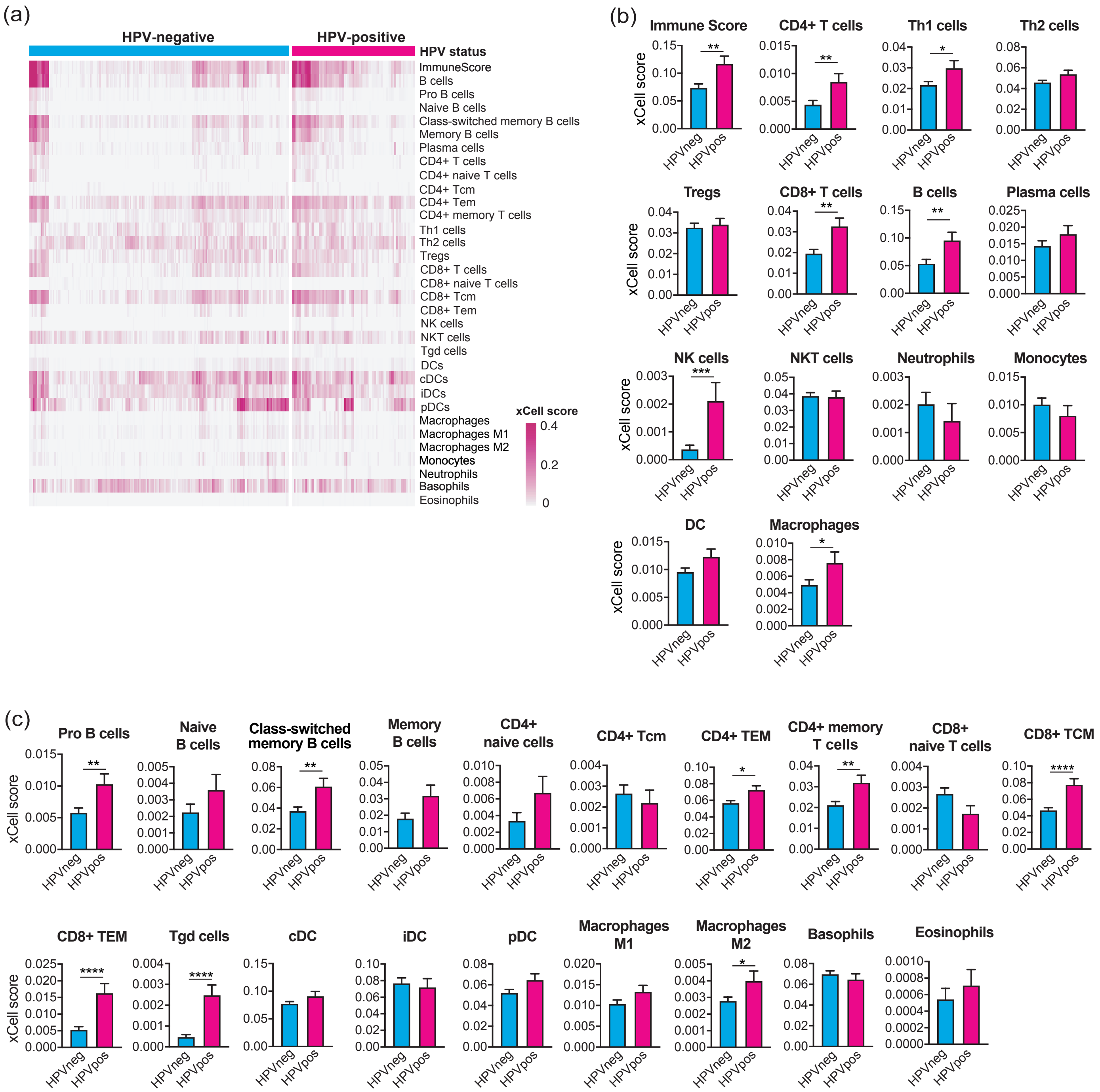

Suppl. Figure 3. Enrichment scores of 33 immune cell types in HNSCC samples

a, Heat map of xCell enrichment scores of 33 immune cell types in 73 HPV-positive HNSCCs and 196 HPV-negative HNSCCs (GSE65858 dataset). b, Bar graphs of enrichment scores of major immune cell types shown in a. c, Bar graphs of enrichment scores of other immune cell types shown in a. HNSCC, head neck squamous cell carcinoma; HPV, human papillomavirus; HPVneg, HPV-negative; HPVpos, HPV-positive; DC, dendritic cells. *, $p < 0.05$; **, $p < 0.01$; ***, $p < 0.001$; ****, $p < 0.0001$.

Suppl.Figure 4

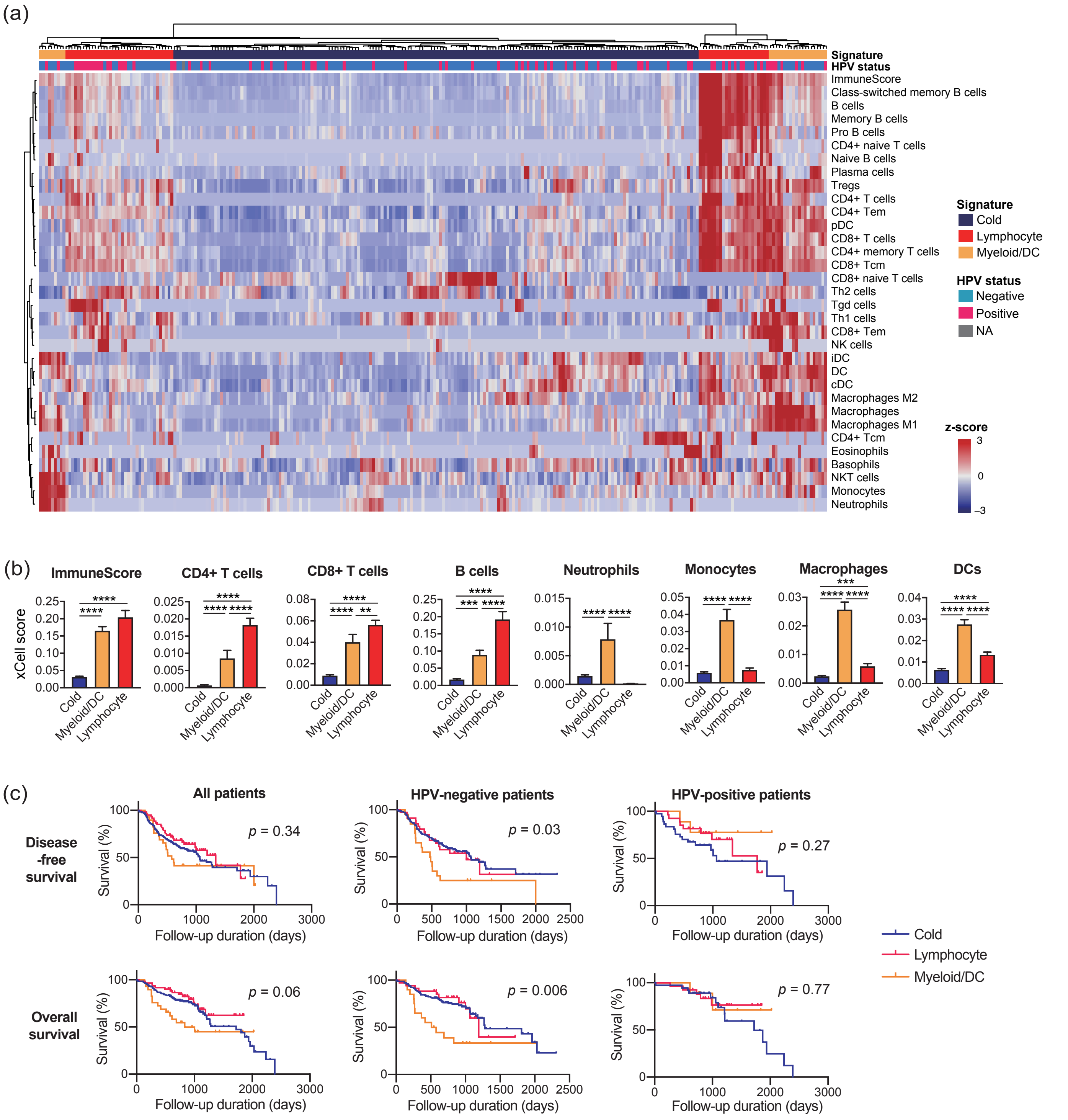

Suppl. Figure 4. Hierarchical clustering of HNSCC samples in GSE65858 dataset

a, Heat map showing non-supervised hierarchical clustering of 270 HNSCCs based on enrichment scores of 33 immune cell types. b, Bar graphs of enrichment scores upregulated in the lymphocyte signature or the myeloid/DC signature. c, Kaplan-Meier survival curves based on the three immune signatures. HNSCC, head neck squamous cell carcinoma; HPV, human papillomavirus; DC, dendritic cells. *, $p < 0.05$; **, $p < 0.01$; ***, $p < 0.001$, ****, $p < 0.0001$.

Suppl.Figure 5

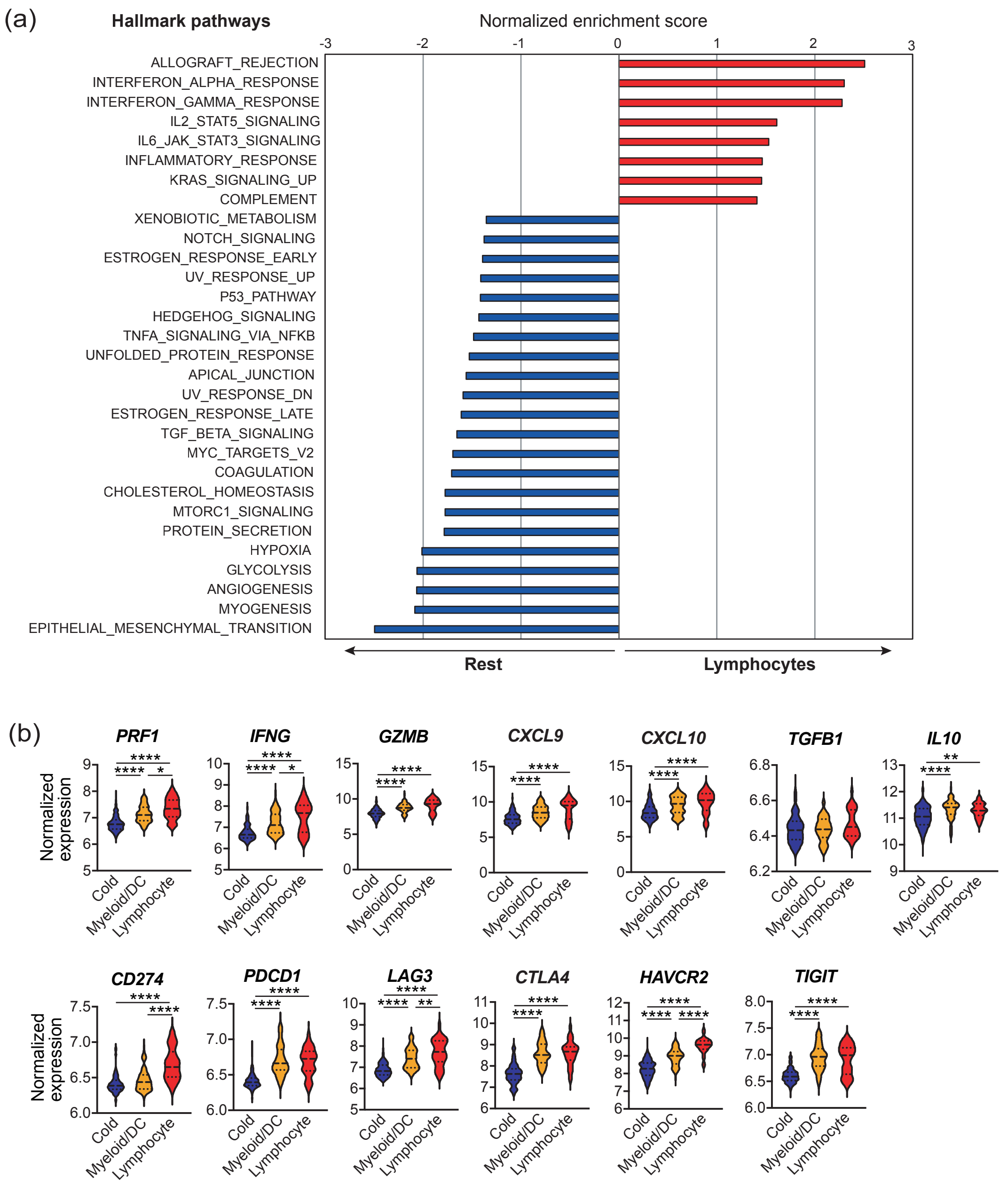

Suppl. Figure 5. The lymphocyte signature correlated with activated cytotoxic T cell response

a, Upregulated and downregulated hallmark pathways in the lymphocyte signature of GSE65858 cohort obtained by GSEA (FDR < 0.05). c, Violin plots of normalized expression of immune-related genes in GSE65858 cohort. GSEA, Gene set enrichment analysis; FDR, false discovery rate. *, $p < 0.05$; **, $p < 0.05$; ***, $p < 0.001$; ****, $p < 0.0001$.
